# Preclinical trials of distant noninvasive electromagnetic therapy for fracture recovery in rats

**DOI:** 10.3389/fvets.2025.1635644

**Published:** 2025-09-25

**Authors:** Irina Victorovna Kuzmina, Ekaterina A. Galkina, Elena Vladimirovna Bondarchuk, Oleg Vyacheslavovich Ovchinnikov, Igor Fedorovich Turkanov, Valery Georgievich Gryaznov, Alexey Gennadievich Vaganov, Grigory Arnoldovich Flaks, Lyubov Germanovna Aistova

**Affiliations:** ^1^Scientific Center of Concern GRANIT, JSC, Moscow, Russia; ^2^Far Eastern State Agrarian University, Blagoveshchensk, Russia

**Keywords:** noninvasive electromagnetic therapy, EMF, PEMF, bone fracture, bone tissue, preclinical trials, rats, morpho-biochemical blood analysis

## Abstract

**Resume:**

To date the injury rate is becoming an acute and significant problem. Very often, injuries are the cause of patient disability and, subsequently, have a lasting negative impact on human health. Among the various types of injuries, bone damages, including fractures of long tubular bones, deserve a special attention.

**Purpose:**

This study evaluates the effect of noninvasive distant electromagnetic therapy on fracture healing in rats.

**Materials and methods:**

The research was performed on the basis of the Russian Federation “Scientific Research Institute of General Pathology and Pathophysiology” on 48 white nonlinear male rats in accordance with the bioethical norms of this institution and the general ethical principles of experiments with animals, consistent with the provisions of the “European Convention for the Protection of Vertebrates Used for Experimental and Other Scientific Purposes” (2003). The animals were divided into two groups. All rats underwent surgery to create a tibial fracture damage without displacement. The groups were disposed in different rooms, located at a considerable distance from each other. A distant noninvasive electromagnetic therapy device was applied for experimental animals with very weak non-ionized non-thermal pulsed electromagnetic fields (PEMF).

**Results:**

Morpho-biochemical blood analysis had shown a decrease in the leukocytes total number, an increase in erythrocytes and platelets for all study stages in the experimental group. And also, a reliable increase in calcium ions in the blood had been detected the last week of the preclinical trials. According to the results of X-ray studies, the bone fractures in the experimental group completely healed a week earlier than in animals of the control group. While in control individuals the recovery process had required 30 days, in animals of the experimental group it had completed after 23 days. The weight gain by animals had demonstrated the general stress reaction development and pain syndrome immediately after surgery and the following gradual recovery and condition stabilization. Weight gain in the experimental group occurred to be weaker vs. the control group that, obviously, associated with a bone fusion accelerated process, which requires a large amount of energy to restore tissue.

**Conclusion:**

Our data indicated a more effective course of wound recovery and bone tissue fusion after surgery with the absence of purulent abscesses under the influence of electromagnetic therapy.

## Introduction

In the modern world, there is a rapid traumatism increase, which is becoming one of the key social problems, leading to disability of a significant part of the population (1). A particular attention should be paid to military conflicts, which are one of the main causes of trauma on a global scale. Wars lead to multiple injuries, disabilities and deaths, and also have a long-term negative impact on the mental and physical health of survivors (2). According to statistics, up to 250,000 people die every year as a result of road accidents. In addition, about 10 million people suffer serious injuries. A significant proportion of injuries to the musculoskeletal system are fractures of long tubular bones (3). Currently, several treatment options are generally accepted, the main goal of them is matching and rigid fixation of bone fragments (4). Conservative measures solve this problem by means of skeletal traction, however, in some cases, this method does not allow the anatomically precise matching of bone structures. The meaning of surgical intervention is open reposition of bone fragments with their fixation with Kirschner wire, plates with limited contact, screws and other metal structures. These methods absolutely ensure matching of bone fragments, but have a number of disadvantages (5). Firstly, it is a surgical operation with associated anesthetic risks; secondly, during these interventions the periosteum is destroyed, disrupting innervation and intraosseous blood supply; thirdly, the imposition of burr holes and using of intramedullary metal structures leads to the destruction of bone marrow and can cause osteomyelitis (6). Modern medicine offers new improved fixing devices, but the essence of the treatment remains the same: rigid, controlled fixation of the fracture site, the opportunity of early patient activation and limbs functional activity. Therefore, the development of methods that accelerate fracture healing, improve local blood supply, which facilitates earlier removal of fixing devices and allows for the prevention of purulent complications, is a pressing issue in modern traumatology. There are many studies in the literature and clinical practice devoted to effects of various therapeutic methods on fracture healing, including ultrasound, shock wave, laser, and electromagnetic therapy (7). Some researchers reported on bone fracture recovery acceleration when exposed to extremely low frequencies of electromagnetic fields (EMF) in the range from 1 to 300 Hz. However, this effect was not unambiguous (8, 9). There are studies in which the authors indicated the absence of any effect or even EMF negative effects on bone tissue regeneration (10). Therefore, the aim of our study is to evaluate the non-invasive remote electromagnetic therapy effect on bone fracture healing in rats.

## Materials and methods

The research was carried out on 48 white nonlinear male rats weighing 230-250 g in the Russian Federation “Scientific Research Institute of General Pathology and Pathophysiology” in accordance with its bioethical norms. Before the operations, the animals were divided into two equal groups - control and experimental. All rats underwent surgery to create a fracture defect of the right hindlimb without displacement of the bones.

All studies were carried out in accordance with the general ethical principles (11). The groups were located in different rooms, standing at a considerable distance from each other (to escape the EMF treatment of the control animals).

All necessary conditions and standards meeting the lab animals maintenance were provided in the premises for the animals of the control and experimental groups. The housing conditions met the requirements of the Russian Federation standards GOST 33215–2014 “Guidelines for the maintenance and care of laboratory animals. Rules of equipment of premises and organization of procedures” and GOST 33216–2014 “Guidelines for the maintenance and care of laboratory animals. Rules for the maintenance and care of laboratory rodents and rabbits.” The sanitary and epidemiological regulations SanPin 2.6.1.2612–10 “Basic Sanitary Rules for Radiation Safety (OSPORB-99/2010),” approved by Resolution No. 40 of the Chief State Environmental Health Officer of the Russian Federation dated April 26, 2010, were also observed. In addition, the radiation safety standards established in SanPiN 2.6.1.2523–09 “Radiation Safety Standards (NRB-99/2009),” approved by Resolution No. 47 of the Chief State Environmental Health Officer of the Russian Federation dated July 7, 2009 and registered by the Ministry of Justice of the Russian Federation by August 14, 2009, were taken into account.

**Figure 1:**
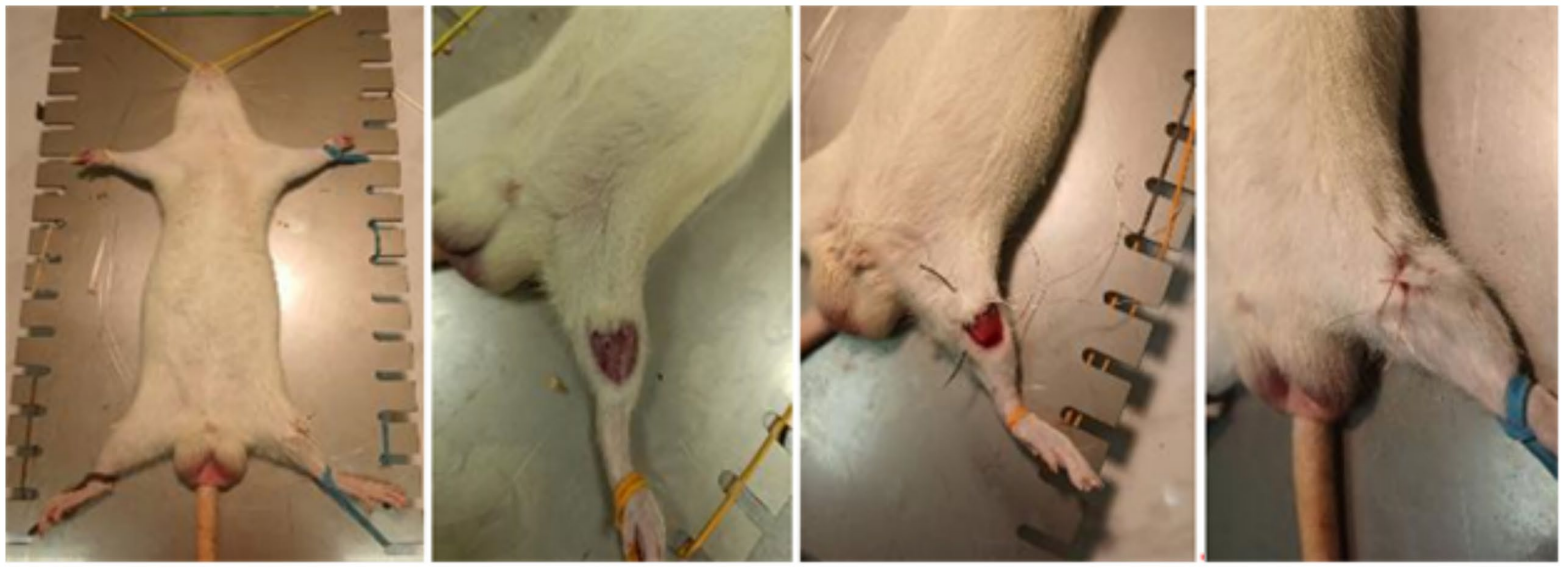
Stages of the operation (from left to right: 1 – fixation of the animal; 2 – bone incision without displacement or formation of fragments; 3 – external appearance of the surgical wound; 4 – the surgical wound is sutured with a knotted suture, non-absorbable material).

All surgical procedures were performed by qualified personnel. After anesthesia of the rats, a 1 cm long surgical discission was made on the right hindlimb. The muscles were spread apart without injury for wider access to the bone. An incision was made in the bone in the area of the tibia proximal epiphysis without displacement or fragments. The surgical wound was then closed with a knotted suture made of non-absorbable material (Figure 1).

After the operations, the animals were kept separately in plastic cage on a crumb’s litters (crumb fraction II – 3 - 6 mm from corn kernels, “Golden Cat” Ltd., Russia). They had free access to feed and water according to requirements of Russian Standard GOST R 50258–92, with an exchange energy of at least 307 Kcal/100 g. Light mode: 12 h — illumination, 12 h — dark, air temperature – in the range 19–25°C, relative humidity — 50–70%.

For the PEMF treatment of animals from the experimental group, the serial non-invasive electromagnetic therapy “TOR” device (Russia) was chosen. It’s using appeared to be effective in the treatment of patients with Sars-Cov-2 (12, 13), and so device was registered as a medical device under number RZN 2021/15459 in the Federal Service for Surveillance in Healthcare of the Russian Federation by 23.09.2021.

The device’s processing principle based on weak non-ionizing non-thermal EMR continuously generated by high-voltage pulses with an amplitude of 5–8 kV, which are capable of affecting the pH of isotonic solutions at a distance of up to 700 m (14). The pulse frequency was 100–150 Hz. Each wave packet with steep rectangular edges (meanders) contained frequency modes 25 kHz-fold. The operating power of the device did not exceed 80 Watt. The collateral damage effects of this technology are not discovered to date. The mode of action of the device was: three times a day (at 20.00, 02.00, 08.00) for 5 min during 14 days.

Blood sampling for analysis and amputation of the hind limbs of the animals was performed weekly throughout the experiment: 6 animals were removed from each group by decapitation. After examination, all the collected paw samples were sent for X-ray examination.

X-ray examinations were conducted at the certified veterinary clinic “VetS V.” The X-ray room is equipped with an Arman 10 L6-01 X-ray machine with a STRIX digitizer (Russia). Paw samples were X-rayed in the lateral projection. The resulting X-ray images were further processed and objective assessment of bone healing by means of criteria of bone fusion, callus formation, and degree of healing were provided.

The bone damage investigations in rats were carried out as the fracture site healed and grew together.

Blood serum biochemical studies for the calcium ion content were also carried out in the same veterinary clinic, samples were immediately delivered for analyzes after blood collection.

The blood count was performed in an automatic hematology analyzer DF-50 (China). Blood was collected in disposable Vacuette vacuum tubes the with the anticoagulant KZ-EDTA.

For data statistical processing, mean value (M) and the standard deviation (±m) were calculated by Excel program. The differences’ reliability was estimated by the Student’s t-test, the differences are considered statistically significant at *p* < 0.05.

## Results

**Figure 2:**
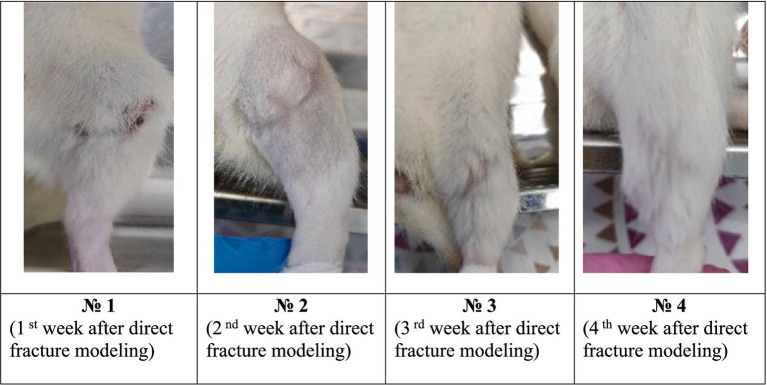
Model direct fracture photos in the control group during the experiment.

**Figure 3:**
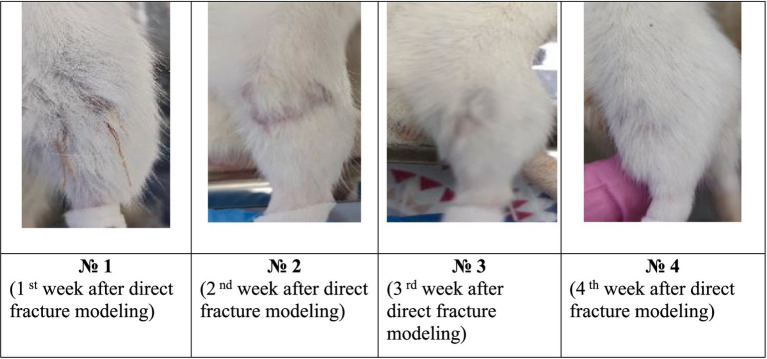
Photographs of the fracture site after surgery in the experimental group during the experiment.

During the analysis carried out by the 6th day after the surgical intervention on modeling the tibia direct fracture, it was found that all the animals form both groups had no tissue edema, and the suture was completely healed (Figures 2, 3). However, in the control group, 67% of the animals had a small scar on the suture surface, while in the experimental group this figure was 17%. This indicates more active recovery and a lower risk of cicatricle tissue formation in the experimental group.

**Figure 4:**
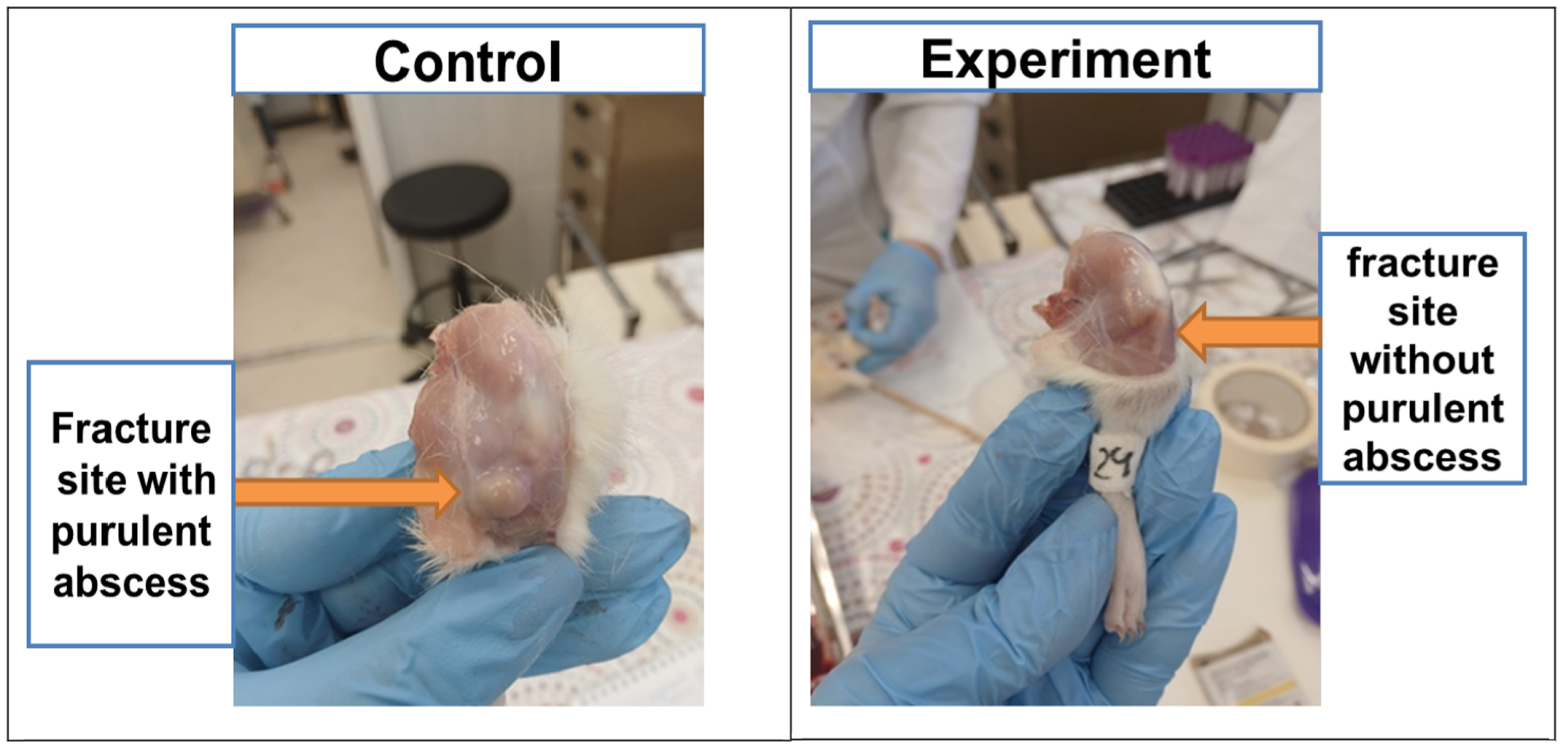
Purulent abscesses in animals of the control group and absence of abscesses in the experimental group.

By the 16 th day after the modeling, tissue edemas were also absent in all animals, and the suture had completely healed. In the control group, 83% of animals developed subcutaneous abscesses, absenting in the experimental group (Figure 4).

By the 23 rd day after the modeling, animals from the control group had a healed scars with a small calluses and cyanotic muscles. In the experimental group, all animals also had a healed scars and calluses of various sizes without any changes in muscle tissues (Figure 3).

The data analysis of the 30th day had shown that all control animals developed small calluses, and the muscles had acquired a bluish tint. In 33% of animals in this group, the muscles remained unchanged. In the experimental group, all animals had healed callosal scars varying size, while in 83% of animals the muscles did not change. This indicates a significant reduction in the risk of infectious complications in the experimental group after the EMF therapy.

X-ray examination results.

While processing radiographic data, it was revealed the destructive changes in the tibia were observed in the images of all the animals, expressed in bone damage without displacement with minor bone fragments near the modeled fracture area.

The results obtained by the 6th day had not revealed any consolidation with a clear visualization of the fracture line in both groups. Structural changes, decreased bone density, and in some cases bone fragments near the fracture were observed.

By the 16th day, all experimental animals had shown clear consolidation signs in the fracture zone and the dense bone callus formation. However, 33.3% among control animals had indicated the active consolidation without forming the bone callus.

**Figure 5:**
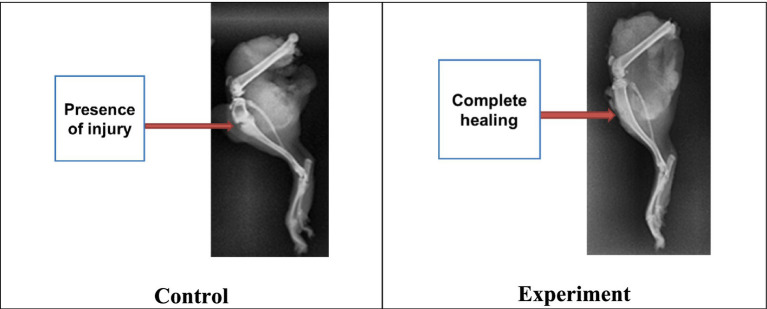
X-ray images of experimental rat tibia by the 23rd day.

By the 21st day, all animals in the experimental group had shown almost complete recovery by primary healing with signs of complete consolidation, without secondary signs of fibrosis formation. In the control group, only one animal had demonstrated primary bone healing with 95% confidence, as well as clear and certain consolidation without weakening in the fracture zone (Figure 5).

**Figure 6:**
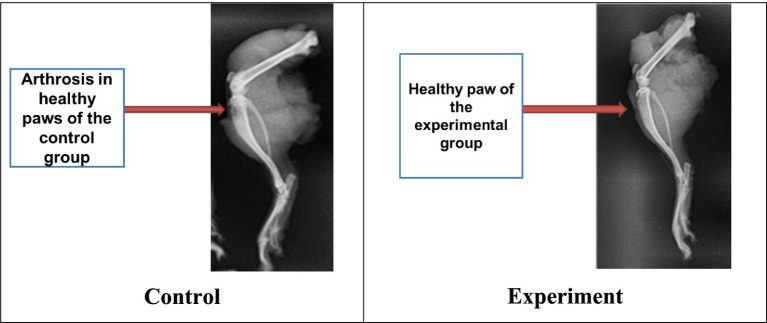
Arthrosis effect in healthy paws of animals both groups.

As a result, it was found that animals from both groups experienced complete consolidation of bone tissue. But this process had occurred a week earlier in the experimental group contrary to the control group. To study the radiographs of limbs with bone tissue damage, healthy limbs of some animals from the experimental and control groups were also examined (Figure 6).

It was noted that most animals from the control group developed minor structural changes in the hindlimb knee joint area (arthrosis), due to the additional load when the other paw was damaged. However, no such pronounced changes were found in animals from the experimental group (Figure 6).

### Animals live weight variation during the experiment

**Figure 7:**
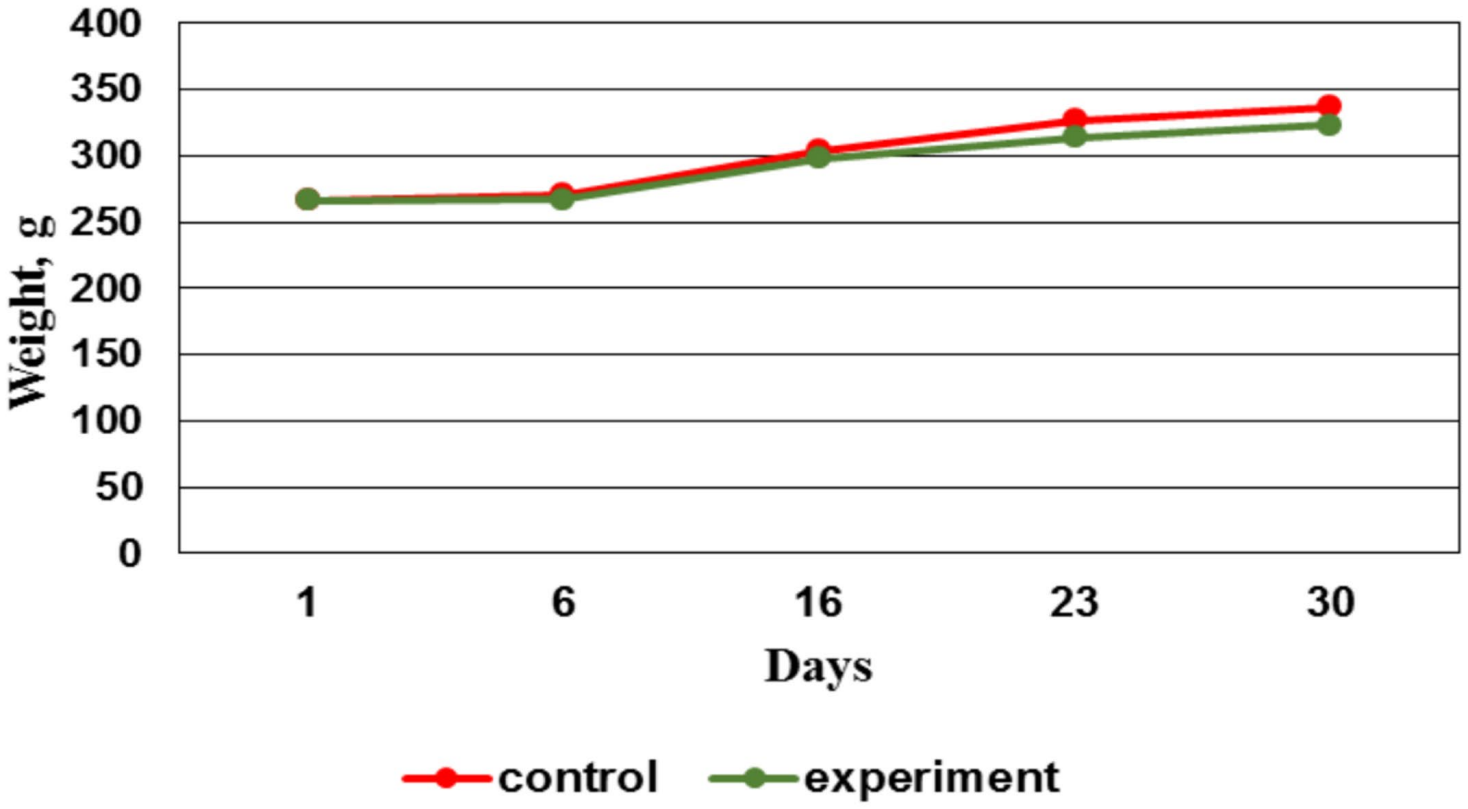
Live weight dynamics by groups, g.

When assessing the animals body weight throughout the experiment, no significant difference in weight indicators was found between the control and experimental groups (Figure 7).

By the 14^th^ day, the EMF therapy was completed. And after that by the 21^st^ day, the weight gain in the control group was more intense vs. the experimental group. This situation persisted until the preclinical trials ending. The less weight gain in the experimental group starting from the 16th day may be due to the accelerated process of bone fusion, which required a large amount of energy to restore tissue. These changes affected the body energy balance, which subsequently led to a decrease in animals body weight in the experimental group.

### Blood morphological parameters studies

**Figure 8:**
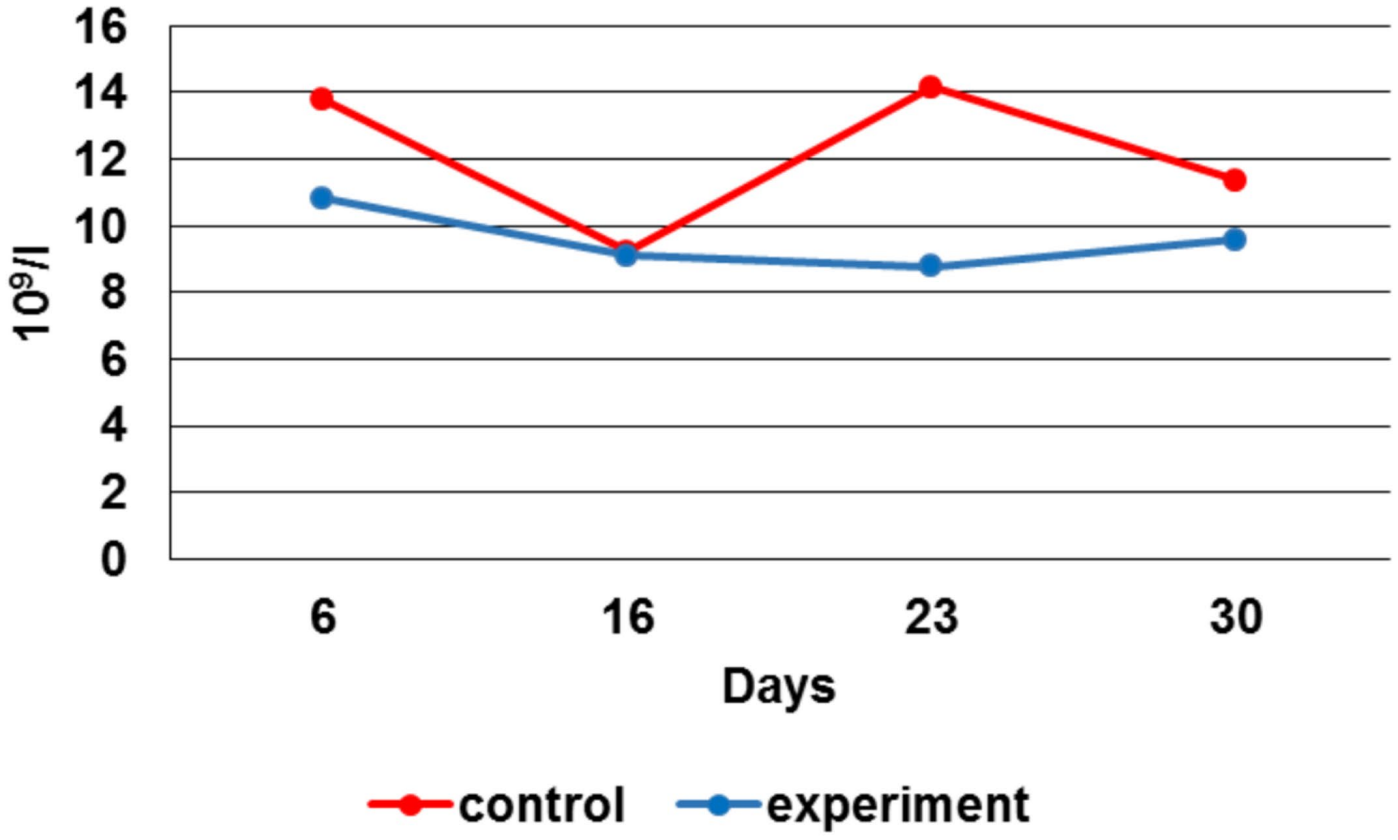
Leukocytes number dynamics.

To assess the animal’s health and identify inflammatory processes after bone fracture modeling the hematological blood tests were performed. Figure 8 displays the total number of leukocytes in the control group, exceeding similar indicators in the experimental group during the study.

**Figure 9:**
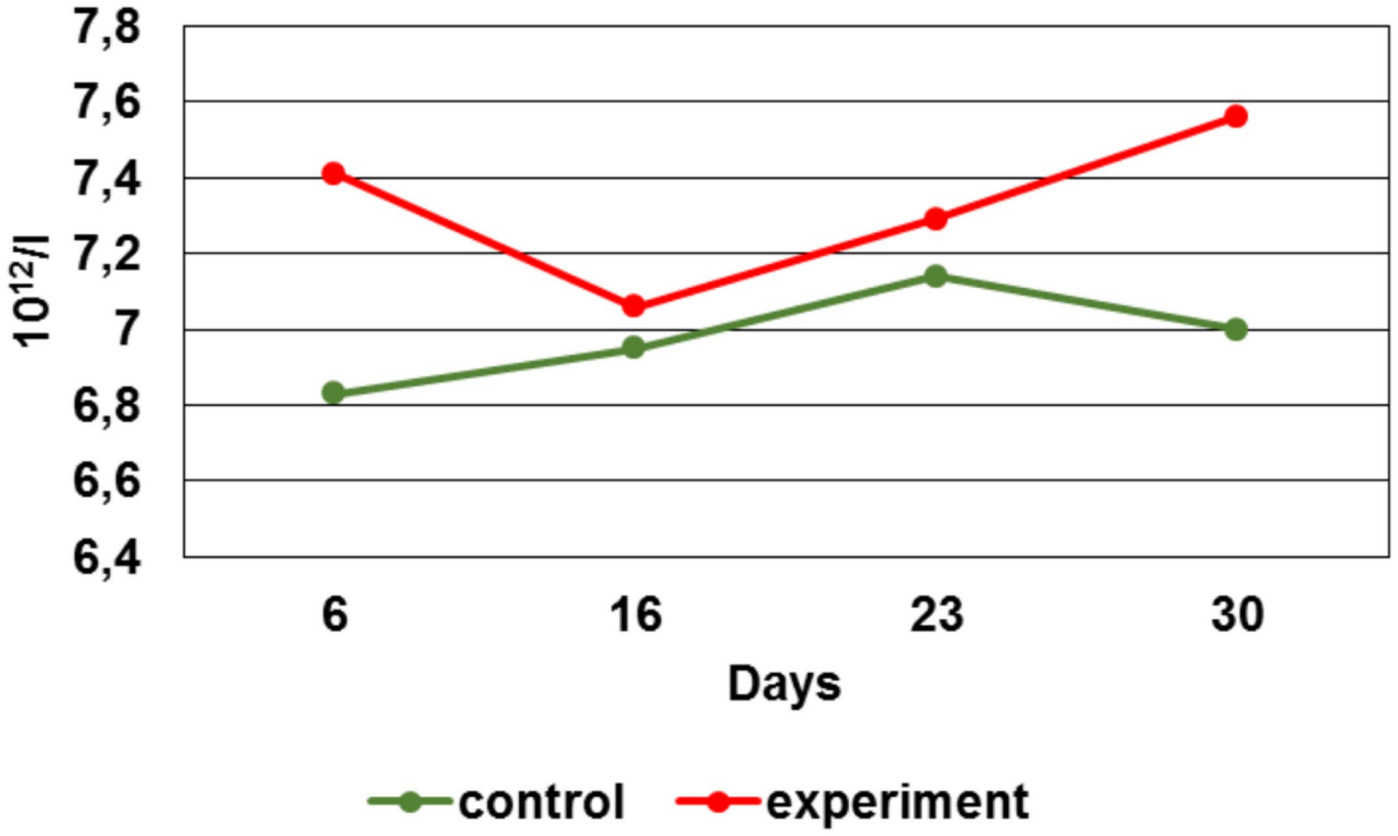
Erythrocytes number dynamics.

By the 6 th day, the leukocytes number in the experimental group was 28% lower, by the 16 th day - 1%, by the 23 rd day — 61%, by the 30 th - 19% vs. the control group. When comparing the erythrocytes number average values during the experiment, their increase in the experimental group can be noted by the 6 th, 23 rd and 30 th day in respect to the control group (Figure 9).

**Figure 10:**
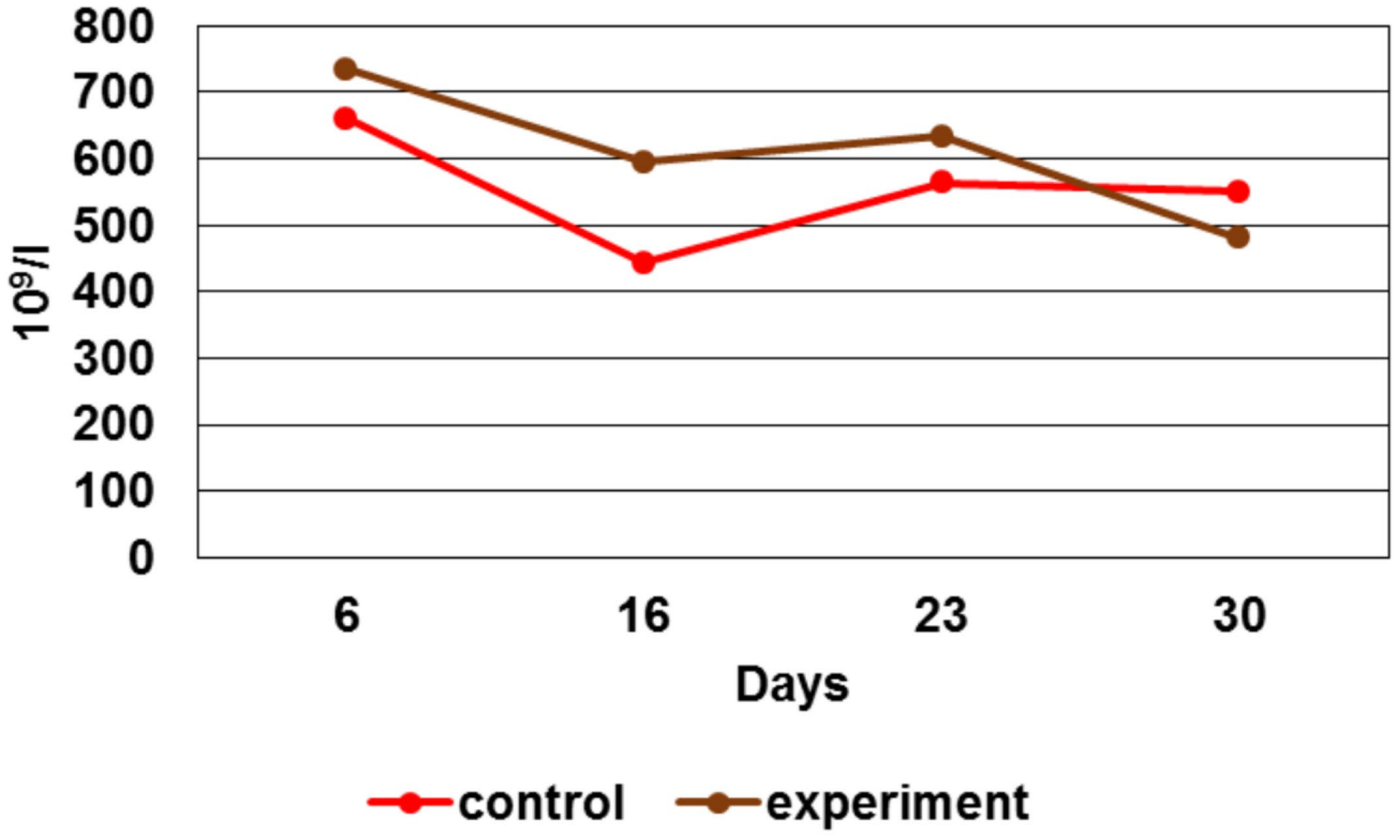
Platelets number dynamics.

Du ring the study, changes in the platelets number were detected (Figure 10).

By the 6 ^th^ day, the platelet number in the experimental group was higher than in the control by 11%, by the 16th day - 34%, by the 23rd day - 12%. However, at the study last stage (day 30th), a platelet number decrease in the experimental group by 15% was recorded. A platelet number increase for the first three stages had indicated the maintenance of an adequate level of coagulation and provision of hemostasis in response to injury.

### Biochemical analyses of calcium ions in rat blood serum

**Figure 11:**
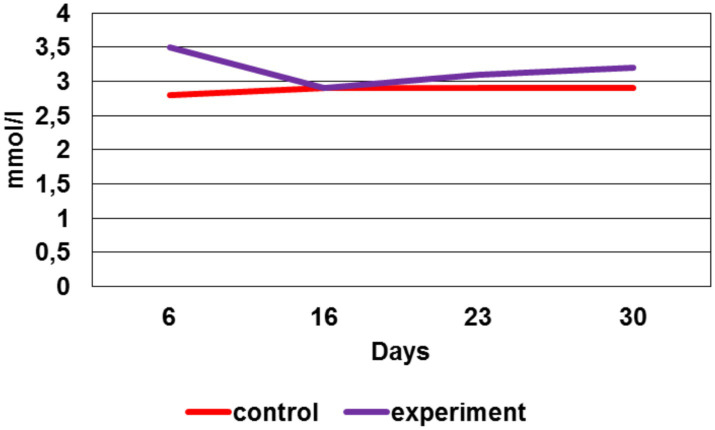
The calcium ion concentration in rat blood serum, mmol/l.

The 4th week after the modeling, it was clear that the calcium ion concentration in the experimental group is significantly higher (*p* < 0.05) compared to the control (Figure 11).

Moreover, the total calcium concentration in the experimental group during the preclinical trials (except for the 2nd week, where its concentration was equal to the control values) was also significantly higher (*p* < 0.05) compared to the control.

## Discussion

Fracture recovery and regeneration bone tissue are rather complicated processes integrating lot of ambient regulated mechanisms both local and general nature. Despite of the serious progress in understanding these processes, there are still unsolved problems required future investigations. Bone tissue recovery is a rather sophisticated multi-channel biological phenomenon, including space–time dependent interactions of different origin cells, extra-cellular matrix components and gene varieties (15). Violation of these phenomena may be caused by many factors as well as high-energy traumas (fragmented fractures, soft tissues crush injuries, open fractures with serious blood -circuit misfunctions and heavy osteogenic tissue damages), mistakes in fracture medication technology and infection complications (16, 17).

Physiological peculiarities such as old ages senile age, chronic diseases, as well as unhealthy habits (smoking, alcoholism, etc.) Body physiological features, such as declining years and senility, including smoking, alcoholism and taking certain medications, can also negatively affect reparative osteogenesis. Combinations of three or more predisposing factors increases the impaired osteotherapy risk, which requires additional early treatment aimed at stimulating bone repair processes.

Over the past 50 years, physical methods of influence to stimulate osteogenesis had been intensively studied. These methods are not specific, but they are more accessible, in most cases non-invasive, do not require special skills, expensive equipment, being characterized by incomparably fewer complications, and at the same time lead to positive results (18).

In the present research, electromagnetic therapy was applied to accelerate bone tissue recovery after fractures. We concluded that this method has several directions of action. Firstly, the skin recovery processes after surgery were accelerated, which is extremely important in clinical practice when performing open repositions of bone fragments. Secondly, in the experimental group, not a single case of suppuration in the area of surgical access to the bone was recorded, which indicates the bactericidal effect of electromagnetic therapy.

Thus, in the absence of pathogenic microflora, a favorable background was originated for the accelerated course of all aseptic inflammation phases. It should be noted that the prime cause mechanism for implementing this effect was the oxidative stress suppression by weak EMFs, which was accompanied by an increase in cellular and tissue destruction due to the release of free oxygen radicals’ large number in the acute inflammation phase. In addition, antioxidant intracellular systems were activated at the mitochondria level. Thus, all inflammation phases were more harmonious and balanced, which directly accelerated regeneration.

Studies show that exposure to electromagnetic fields and waves of various frequencies affects the activity of key enzymes, mitochondrial function, the level of energy metabolic production and oxidative stress. Thus, exposure to electromagnetic radiation increases the activity of antioxidant enzymes such as catalase and superoxide dismutase in bacteria such as staphylococcus, intestinal bacilli and *pseudomonas aeruginosa*, reaching a maximum after about 45 min of exposure. This effect serves as an important mechanism for protecting microorganisms from oxidative stress and their survival in conditions of intraorgan oxidative processes (19). At the cellular level, high-frequency radiation in the THz range has a direct effect on mitochondria. It inhibits the respiratory chain by approximately 30%, and at a higher power density (up to 0.7 kV/cm) - by two times, which leads to a significant decrease in the production of ATP - the main energy carrier of the cell (20, 21). Along with this, an increase in the permeability of mitochondrial membranes is observed, which indicates their disruption - probably due to electroporation or the opening of non-specific conductivity pores, such as mitochondrial mPTP pores. In addition, the presence of calcium in the medium causes swelling of mitochondria and promotes the opening of these pores, which, in combination with an increase in the production of reactive oxygen species (ROS), initiates lipid peroxidation and increased destruction of mitochondrial membranes (22–25). These processes lead to a decrease in the proton potential, dysfunction of the energy chain and a decrease in ATP synthesis, which emphasizes the importance of the balance of ion channels and oxidative stress for cell functioning.

The inflammation aseptic nature in the post-surgery wound had a positive effect on bone regeneration. On the one hand, this helped to avoid infection in the fracture area, and on the other hand, there was inflammatory reactions decrease due to diminishing the bacterial load. In our preclinical trials, this was confirmed by the hematological tests, in which the leukocytes level in the experimental group remained within the norms or slightly increased.

At the same time, moderate leukocytosis was observed in the control group. The decrease in the body weight gain of the experimental animals in combination with erythrocytosis indicated the intensity and energy consumption of bone tissue regeneration processes in the experimental group. The acceleration of bone tissue healing processes was indirectly confirmed by X-ray images, which indicated complete consolidation at the fracture sites in the experimental group by the 23rd day, while in the control group by this time the healing process continued until the end of the experiment. The calcium ion level increase indirectly supported the EMF therapy influence on the humoral regulation of mineral metabolism during bone tissue regeneration processes, as well as on the harmonious work of osteoblasts and osteoclasts directly in the fracture area.

At present, the mechanisms underlying the therapeutic electromagnetic effects, as well as their dependence on the spectra, intensity, exposition time, periodicity etc., remain insufficiently studied. Therefore, EMF therapy investigations in this area will be continued.

## Conclusion

Thus, the obtained data objectively indicate the positive effect of remote non-invasive electromagnetic therapy on bone regeneration processes, accelerating recovery by a week, and demonstrate a potentiality to apply as an auxiliary treatment along with classical methods of bone repositioning.

## Data Availability

The datasets presented in this study can be found in online repositories. The names of the repository/repositories and accession number(s) can be found in the article/supplementary material.
